# COVID-19 in acute myeloid leukemia (AML) and myelodysplastic syndrome (MDS): a propensity matched analysis (2020-2021)

**DOI:** 10.3389/fonc.2024.1446482

**Published:** 2024-10-17

**Authors:** Barath Prashanth Sivasubramanian, Shashvat Joshi, Diviya Bharathi Ravikumar, Madhumithaa Jagannathan, Sonia Babu, Shanthi Reddy Sripathi, Avinash Javvaji, Priyanshu Jain, Dinesh Kumar Shanmugam, Bharath Duraisamy Swami Kannan, Raghavendra Tirupathi, Rutul Dalal

**Affiliations:** ^1^ Northeast Georgia Medical Center, Gainesville, GA, United States; ^2^ Shanghai Medical College, Fudan University, Shanghai, China; ^3^ ESIC Medical College and Postgraduate Institute of Medical Science and Research, Chennai, Tamil Nadu, India; ^4^ Maharashtra Institute of Medical Education and Research (M.I.M.E.R) Medical College, Talegaon Dabhade, Pune, Maharashtra, India; ^5^ M.S Ramaiah Medical College, Bangalore, Karnataka, India; ^6^ Osmania Medical College, Hyderabad, Telangana, India; ^7^ Chalmeda Anandrao Institute of Medical Sciences, Karimnagar, Telangana, India; ^8^ Kasturba Medical College, Manipal, Udupi, Karnataka, India; ^9^ PSG Institute of Medical Sciences and Research, Peelamedu, Coimbatore, Tamil Nadu, India; ^10^ Government Sivagangai Medical College, Sivaganga, Tamil Nadu, India; ^11^ Department of Infectious Diseases, Keystone Health, Chambersburg, PA, United States; ^12^ Medical Director, Infectious Diseases, Penn State Health (Eastern Region), Penn State Health St. Joseph Medical Center, Reading, PA, United States

**Keywords:** AML, myelodysplastic syndrome, COVID-19, severe sepsis, ARDS, acute respiratory failure, MDS, acute myeloid leukemia and HSCT

## Abstract

**Background:**

By 2023, COVID-19 had caused 6.8 million deaths in the United States. COVID-19 presents more severely in leukemia compared to solid tumors (OR 1.6, p<0.05). However, data on Acute Myeloid Leukemia (AML) and Myelodysplastic Syndrome (MDS) are limited. We investigated the mortality in AML and MDS patients with COVID-19.

**Methods:**

Data from the 2020-2021 National Inpatient Sample was used to conduct a cross-sectional analysis. We identified AML and MDS patients with COVID-19 hospitalizations through ICD-10 codes. Analysis was done by propensity matching and multivariate regression with a p-value of ≤0.05.

**Results:**

Of 28,028 AML admissions, 336 (1.2%) were admitted for COVID-19. AML-COVID-19 cohort had a lower hospitalization risk (aOR 0.3, p=0.000) and higher mortality (21.7% vs 8.7%; aOR 1.6, p=0.023) than AML patients admitted for other causes. AML patients post-HSCT (Hematopoietic Stem Cell Transplantation) had a higher risk of COVID-19 (20.2% vs 9.8%; aOR 2.6, p=0.000) and increased mortality (19.1% vs 6.7%; aOR 4.1, p=0.000) compared to other causes. Similarly, of 28,148 MDS patients, 769 (2.7%) were admitted for COVID-19. The MDS-COVID-19 cohort had a lower hospitalization risk (aOR 0.59, p=0.000) and higher mortality (19.6% vs 6.6%; aOR 2.2, p=0.000) compared to other causes. In MDS, HSCT did not alter the risk of COVID-19 hospitalizations (3% vs 3.9%; aOR 0.9, p=0.662), but these patients had higher mortality (17.4% vs 5.1%; aOR 4.0, p=0.032).

**Conclusion:**

COVID-19 hospitalization was low in AML and MDS but carried a high mortality risk. Post-HSCT, the mortality is high, warranting research into understanding the underlying factors.

## Introduction

As of 2023 in the United States, the COVID-19 pandemic has caused over 6.8 million fatalities (1). Immunocompromised patients (those with HIV, transplants, and malignancies) have an increased risk of acquiring COVID-19 infections, developing prolonged infections, and potentially contributing to the emergence of concerning viral variants (2, 3). In hematological malignancies, specifically, those with leukemia are known to experience more severe COVID-19 outcomes compared to those with solid organ tumors (OR 1.57, p<0.0043) (4, 5). Additionally in hematological malignancies, male gender, pre-induction and induction phases, ICU admission, low levels of oxygen saturation at the onset of infection, Rhesus (RH) factor positivity, and higher fibrinogen levels have all been associated with increased mortality. The state of the current malignancy, recent administration of myeloablative chemotherapy or immunosuppressive therapies, recent surgery, and radiotherapy further impact the outcomes (5, 6).

Acute Myeloid Leukemia (AML) patients had a lower COVID-19 prevalence of 5.4% compared to Myelodysplastic Syndrome (MDS) with COVID-19 at 19.7% (6). In patients with AML and MDS, a heightened risk of mortality from COVID-19 is noted, with rates reaching up to 40% and 42.3%, respectively (7–9). Several underlying factors may exacerbate the severity of COVID-19 in hematological malignancies. These include the aggressive nature of their underlying disease, immune dysfunction (particularly involving neutrophils and T-cells), delayed seroconversion, and prolonged hospitalization (10–14). Additionally, treatment outcomes in MDS patients can be challenging, with a high risk of treatment failure (HR 3.564, p=0.041) observed when using antivirals and anti-spike monoclonal antibodies (MABs) for COVID-19 (15). In patients with AML and MDS, those undergoing hematopoietic stem cell transplant (HSCT) have shown mortality rates of 24.8% and 27% for allogeneic (allo-HSCT) and autologous (auto-HSCT) transplants, respectively, following COVID-19 infection (7). Furthermore, AML patients post-HSCT have shown several mutations in the virus, including substitution (D796H) in the S2 subunit, deletion [deltaH69/deltaV70] in the S1 N-terminal domain of the spike protein, E340K, K356R, R346T, and E484V). These mutations have led to viral evolution, a protracted period (4 months) for neutralizing antibodies to appear, and reduced sensitivity to neutralizing antibodies (16–19).

Currently, available studies on AML and COVID-19 are restricted to small cohorts and case reports/series (4). Moreover, evidence-based guidelines to aid clinicians in treatment decisions are scarce due to evolving recommendations for COVID-19 treatment. Additionally, crucial information regarding readmission rates and out-of-hospital mortality rates remains lacking (8, 20).

Our study aims to assess mortality in AML and MDS patients hospitalized with COVID-19. Furthermore, our secondary objective was to assess the outcomes of COVID-19 in AML and MDS patients who have received HSCT.

## Methods

### Design and data source

We queried the 2020-2021 National Inpatient Sample (NIS) database. A cross-sectional study was carried out using the NIS database. The NIS was developed by the Healthcare Cost and Utilization Project (HCUP) and sponsored by the Agency for Healthcare Research and Quality (AHRQ) (21). Its purpose was to generate regional and national estimates of inpatient utilization, expenditures, and outcomes in the United States. The document comprises various components, including patient demographics (such as age, sex, and race), diagnosis and procedure codes derived from the International Classification of Diseases, Tenth Revision, Clinical Modification/Procedure Coding System (ICD-10-CM/PCS), measures of severity and comorbidity, hospital characteristics, discharge status, and length of stay (LOS).

### Ethical consideration, sample size, and study population

The NIS is a de-identified, publicly accessible database. The current investigation was not submitted to an institutional review board for approval. A predetermined sample size was not calculated for this study. Adults aged 18 or above admitted with an ICD10 code of hematological malignancies (acute myeloid leukemia and myelodysplastic syndrome) were included. We followed a method previously used to identify patients with a diagnosis of hematological malignancies (acute myeloid leukemia and myelodysplastic syndrome) and COVID-19 adequately. ICD-10-CM/PCS codes are provided in the **Supplementary Table S1**. We complied with the AHRQ’s data user agreement before accessing the NIS databases. The databases utilized adhere to the HIPAA (Health Insurance Portability and Accountability Act) Privacy Rule’s definition of limited data sets and do not comprise any explicit identifiers of patients.

### Variables assessed

Gender was delineated as male and female. Patient race was defined as White, African American, Hispanic, Asian or Pacific Islander, and Native American. Insurance status was defined by Medicare (referent), Medicaid, Private Insurance, and other/self-pay/No charge. The comorbidities were classified using ICD-10-CM codes. The codes encompass acute kidney injury, myocardial infarction, invasive ventilation, severe sepsis, acute respiratory failure, acute respiratory distress syndrome, acute heart failure, and vasopressor usage. The codes are provided in the **Supplementary Table S1**. The inpatient mortality, total hospital charges, and length of stay were acquired from the NIS database.

### Statistical methods

We conducted our analysis by examining continuous variables through means and t-tests, and qualitative variables were assessed using the chi-square test. We set a significance level of p≤ 0.05. Stata v18 was used to conduct the analysis. We used two different methods to adjust for confounders in our analysis: Propensity score matching and multivariate regression analysis. Propensity scores were used to match AML and MDS patients with COVID-19. A non parsimonious multivariate logistic regression model was developed to estimate the propensity score for developing mortality using the following variables: age, gender, and race. The double robust method was then used to generate treatment weights and the inverse probability of treatment weighting was used to match cases with controls using generalized linear models (22). The second analysis used multivariable regression analysis models to adjust the results for potential confounders. Multivariable regression models were built by including all confounders significantly associated with the outcome on univariate analysis with a cutoff p-value of 0.2. Variables that were deemed important determinants of the outcomes based on literature were added to the models.

### Outcomes

Our primary objective was to determine the factors associated with in-hospital mortality among adults (≥18 years) with AML and MDS, primarily admitted for COVID-19. Our secondary objective was to understand the mortality of COVID-19 in patients who are HSCT recipients. Additionally, we studied the healthcare utilization (length of stay and total charges incurred) and the prevalence of comorbidities in hospitalized adult AML/MDS patients with COVID-19.

## Results

### Acute myeloid leukemia with COVID-19

A total of 28,028 AML patients were identified, with 337 patients (1.2%) hospitalized for COVID-19. This rate was lower compared to a 4.6% hospitalization rate among 11 million non-AML cases (p<0.001). **Supplementary Table S2** represents the sociodemographic profile of Acute Myeloid Leukemia Patients. The average age of patients did not differ between the two groups (62.3 ± SE 0.9 vs 61 ± SE 0.1, p=0.1919, non-significant). **Table 1** represents the outcomes of Acute Myeloid Leukemia (AML) patients who were hospitalized for COVID-19. **Supplementary Table S3** represents univariate logistic regression of AML patients and COVID-19 who did not survive. AML patients showed a decreased risk of COVID-19 hospitalization compared to AML patients admitted for other reasons (adjusted Odds Ratio aOR of 0.3; 95% CI 0.2-0.3; p<0.001) when adjusted for age over 65, gender, race, hospital characteristics (size, teaching status, and location), complications and comorbidities (stroke, acute respiratory distress syndrome, acute respiratory failure, sepsis, acute kidney injury, and myocardial infarction), and interventions (vasopressor usage and invasive ventilation).

**Table 1:** Outcomes of Acute Myeloid Leukemia (AML) patients with COVID-19.

Acute Myeloid Leukemia (AML)	All Cause Admissions or Non-COVID-19 Admissions (%)	95% Confidence Interval		COVID-19 Admissions (%)	95% Confidence Interval	
		**Lower Limit CI**	**Upper Limit CI**		**Lower Limit CI**	**Upper limit CI**
Mortality rate	8.7	8.3	9.0	21.7	17.5	26.6
**COVID-19 Risk in Acute Myeloid Leukemia (AML)**	**Adjusted Odds Ratio**			p-value		
Hospitalization risk	0.3	0.2	0.3	<0.001*		
Mortality risk	1.6	1.1	2.6	0.028**		
**Acute Myeloid Leukemia (AML) Patients who have received HSCT**	**All Cause Admissions or Non-COVID-19 Admissions (%)**			**COVID-19 Admissions (%)**		
Mortality rate	19.1	11.3	30.4	6.7	5.8	7.7
**COVID-19 Risk in Acute Myeloid Leukemia (AML) Patients who have received HSCT**	**Adjusted Odds Ratio**			**p-value**		
Hospitalization risk	2.6	1.9	3.4	<0.001***		
Mortality risk	4.1	2.0	8.7	<0.001****		

AML stands for Acute Myeloid Leukemia patients, COVID-19 admissions represent AML patients who were admitted for COVID-19 as the primary diagnosis, All cause Admissions or Non-COVID-19 Admissions represent AML patients who were admitted for diagnosis other than COVID-19, HSCT stands for Hematopoietic stem cell transplantation.

* were matched for age, gender, and race based on multivariate regression adjusted for age over 65 years, gender, race, hospital characteristics (size, teaching status, and location), comorbidities and complications (stroke, sepsis, ARDS, acute respiratory failure, acute kidney injury, and acute heart failure), and interventions (vasopressor usage and HSCT).

** were matched for age, gender, and race based on multivariate regression adjusted for age over 65 years, gender, race, hospital characteristics (size, teaching status, and location), comorbidities and complications (stroke, sepsis, ARDS, acute respiratory failure, acute kidney injury, and acute heart failure), and interventions (vasopressor usage and HSCT).

*** were matched with age over 65 years, gender, and race based on multivariate regression adjusted with age (≥65 years), gender, race, and hospital characteristics (size, teaching status, and location).

**** were matched with age over 65 years, gender, and race based on multivariate regression adjusted for age(≥65 years), gender, race, and hospital characteristics (size, teaching status, and location).

The mortality rate in AMLCov was 22.7% (95%CI 16.4%-30.4%) while in All-cause-admission was only 8.8% (95%CI 8.3%-9.4%). Among these, patients aged ≥65 years showed a high mortality rate (Nonsurvivors 74% vs Survivors 48.1%, p=0.0001). Male patients experienced high mortality (Nonsurvivors 60.3% vs Survivors 49.4%, p=0.1028, non-significant). Additionally, based on a median household income of $1 - $49,999, we observed a higher number of nonsurvivors than survivors (33.3% vs 27.3%, p=0.5907, non-significant). We observed significantly higher occurrences of severe sepsis (26% vs 1.9%, p<0.0001), acute respiratory distress syndrome (27.4% vs 3%, p<0.0001), acute respiratory failure (65.7% vs 52.3%, p=0.0459), and myocardial infarction (12.3% vs 1.5%, p<0.0001) among nonsurvivors during hospitalization. Additionally, the usage of vasopressors (17.8% vs 1.1%, p<0.0001) and the necessity for invasive ventilation (2.7% vs 0.4%, p=0.056) were markedly higher among nonsurvivors.

**Table 2** represents Comorbidities of Acute Myeloid Leukemia and Myelodysplastic Syndrome patients with COVID-19. After matching with age, gender, and race and adjusting the multivariate regression analysis for age over 65, gender, race, hospital characteristics (size, teaching status, and location), complications and comorbidities (stroke, ARDS, acute respiratory failure, sepsis, acute kidney injury, and myocardial infarction), and interventions (vasopressor usage, invasive ventilation, and HSCT status), COVID-19 showed an increased risk of mortality (aOR 1.6; 95% CI 1.1-2.6; p=0.028) compared to non-COVID-19 admissions. We identified several mortality predictors with p<0.05. Age ≥65 years (aOR 2.8; 95% CI 1.3-6.0), severe sepsis (aOR 6.7; 95% CI 1.5-29.5), ARDS (aOR 17.0; 95% CI 3.6-80.8), acute respiratory failure (aOR 4.5; 95% CI 1.5-13.7), myocardial Infarction (aOR 8.4; 95% CI 2.2-32.6), and invasive ventilation (aOR 9.8; 95% CI 1.3-74.9) were found to have a high risk of mortality.

**Table 2:** Comorbidities of Acute Myeloid Leukemia and Myelodysplastic Syndrome patients with COVID-19.

	Acute Myeloid Leukemia with COVID-19 (n=337)			Myelodysplastic Syndrome with COVID-19 (n=769)		
	Survival Percentage (%)	Mortality Percentage (%)	p-value	Survival Percentage (%)	Mortality Percentage (%)	p-value
**Stroke**	0.4	6.9	**0.0002**	0.3	2	**0.0227**
**Opportunistic Infection**	1.1	2.7	0.3192	1	1.3	0.7016
**Severe Sepsis**	1.9	26	**<0.0001**	0.6	17.9	**<0.0001**
**ARDS**	3	27.4	**<0.0001**	1.3	21.8	**<0.0001**
**Acute Respiratory Failure**	52.3	65.7	**0.0459**	49.5	66.9	**<0.0001**
**Acute Kidney Injury**	22.7	45.2	**0.0002**	29.1	45.7	**<0.0001**
**Arrhythmia (Ventricular fib, Ventricular tachycardia, Atrial fib, Atrial flutter)**	6.1	9.6	0.2922	17.5	17.9	0.9064
**Acute Heart Failure and Acute Pulmonary Edema**	4.2	8.2	0.1605	16.2	25.8	0.0062
**Myocardial Infarction**	1.5	12.3	**<0.0001**	4.5	11.3	0.0012
**Sudden Cardiac Arrest**	0	12.3	**<0.0001**	0.2	10.6	**<0.0001**
**Cardiogenic Shock**	0.4	0	0.5985	0.3	0	0.4838
**Diabetes**	33.7	35.6	0.7667	37.1	29.1	0.0706
**Hypertension**	7.6	6.9	0.8326	15.7	17.2	0.642
**Blood Transfusion**	12.9	24.7	0.0138	16	19.2	0.3462
**Vasopressor**	1.1	17.8	**<0.0001**	0.2	9.3	**<0.0001**
**Invasive Ventilation**	0.4	2.7	0.056	0	3.3	**<0.0001**

On analyzing the outcomes in AML patients post-hematopoietic stem cell transplantation (HSCT), we found that a higher proportion of patients were admitted for COVID-19 compared to those admitted for other causes (20.2% vs 9.8%; p<0.001). After matching with age over 65 years, gender, and race and adjusting the multivariate regression analysis for age over 65, gender, race, and hospital characteristics (size, teaching status, and location), the hospitalization risk in the HSCT patients was higher with an aOR of 2.6 (95% CI 1.9-3.4; p<0.001). The mortality rate for COVID-19 was higher (19.1%; 95% CI 11.3%-30.4%) than the non-COVID-19 group (6.7%; 95% CI 5.8%-7.7%). After matching and adjusting with the same variables as before, multivariate regression showed a high mortality risk in COVID-19 with an aOR of 4.1 (95% CI 2-8.7; p<0.001). **Table 3** represents the results of the multivariate regression analysis used to study the predictors of mortality in AML patients with COVID-19.

**Table 3:** Multivariate Logistic Regression to determine predictors of mortality in Acute Myeloid Leukemia patients with COVID-19.

Predictors of Mortality		95% Confidence Interval		
	Adjusted Odds Ratio	Lower Limit CI	Upper Limit CI	p-value
**Age ≥65 years**	2.8	1.3	6.0	**0.007**
**Female Gender**	0.9	0.4	2.0	0.891
**Location/teaching status of hospital (Ref=Rural)**				
Urban non-teaching	0.3	0.1	1.3	0.11
Urban teaching	0.5	0.1	1.9	0.325
Hospital Region (Ref=Northeast)				
Midwest	0.5	0.2	1.4	0.174
South	0.9	0.4	2.3	0.859
West	0.7	0.2	2.2	0.553
**Stroke**	3.1	0.4	23.0	0.272
**Severe Sepsis**	6.7	1.5	29.5	**0.012**
**ARDS**	17.0	3.6	80.8	**<0.001**
**Acute Respiratory Failure**	4.5	1.5	13.7	**0.007**
**Acute Kidney Injury**	1.6	0.7	3.7	0.263
**Myocardial Infarction**	8.4	2.2	32.6	**0.002**
**Vasopressor Usage**	3.2	0.5	19.2	0.205
**Invasive Ventilation**	9.8	1.3	74.9	**0.028**
**HSCT status**	0.5	0.2	1.2	0.118

**Table 4** shows hospital utilization in Acute Myeloid Leukemia (AML) and Myelodysplastic Syndrome (MDS) patients who were admitted for COVID-19. After matching and adjusting with age, gender, race, and hospital characteristics (size, teaching status, and location), we identified the length of hospital stay and hospital charges for AML patients hospitalized for COVID-19. Both of these were lower in COVID-19 compared to non-COVID-19 admissions (Hospital Stay: Coefficient -1.7 days, 95% CI -2.9 to -0.4 days, p=0.011; Charges: Coefficient $ -34,200; 95% CI $-62,200 to $-6,163; p= 0.017).

**Table 4:** Hospital Utilization by Acute Myeloid Leukemia (AML) and Myelodysplastic Syndrome (MDS) patients who were hospitalized for COVID-19.

Use of Hospital Resources comparing patients admitted for COVID-19 vs All-other-admissions	Length Of Stay (in days) Adjusted Mean Difference with 95% Confidence interval	p-value	Hospital Charges (in $) Adjusted Mean Difference with 95% Confidence Interval	p-value
**Acute Myeloid Leukemia**	-1.7 (-2.9 to -0.4)	0.011*	-34,200 (-62,200 to -6,163)	0.017*
**Myelodysplastic Syndrome**	1.26 (0.67 to 1.85)	<0.001*	6,230 (-12,405 to 24,865)	0.512*

Length of stay represents the time patients spent in the hospital after admission until discharge, Hospital Charges represent the total charges incurred by the patients during their entire stay in the hospital, Use of hospital resources represents the length of stay and hospital charges incurred by both AML and MDS patients who got COVID-19 vs their non-COVID-19 or All-cause mortality counterparts.

*Matched for and adjusted with age, gender, race, and hospital characteristics (size, teaching status, and location).

### Myelodysplastic syndrome with COVID-19

In MDS patients (n=28,148), we identified 769 COVID-19 admissions (MDSCov 2.7%), and the remaining were admitted for other reasons. In the non-MDS population of 11 million, we identified 500,000 COVID-19 admissions (4.6%) with a p-value of 0.000. **Supplementary Table S4** represents the sociodemographic profile of patients with Myelodysplastic Syndrome.

The average age of MDSCov (n=769) was higher than those admitted for all other causes (77.3 ± SE 0.4 years vs 75.3 ± SE 0.1 years, p<0.0001). **Table 5** represents outcomes in Myelodysplastic syndrome (MDS) patients who were hospitalized for COVID-19. **Supplementary Table S5** represents univariate logistic regression of Myelodysplastic Syndrome patients and COVID-19 who did not survive. The MDS population had a decreased risk of hospitalization for COVID-19 with aOR of 0.59 (95% 0.54-0.63; p<0.001) compared to those MDS patients admitted for other reasons when adjusted for age over 65 years, gender, race, and hospital characteristics (size, teaching status, and location), comorbidities and complications (stroke, sepsis, ARDS, acute respiratory failure, acute kidney injury, and acute heart failure), and vasopressor usage.

**Table 5:** Outcomes of Myelodysplastic syndrome (MDS) patients with COVID-19.

Myelodysplastic Syndrome (MDS)	All Cause Admissions or Non-COVID-19 Admissions (%)	95% Confidence Interval		COVID-19 Admissions (%)	95% Confidence Interval	
		Lower limit CI	Upper limit CI		Lower limit CI	Upper limit CI
Mortality rate	6.6	6.3	7.0	19.6	17.0	22.6
**COVID-19 Risk in Myelodysplastic Syndrome (MDS) patients**	**Adjusted Odds Ratio**			**p-value**		
Hospitalization risk	0.59	0.54	0.63	p= <0.001*		
Mortality risk	2.2	1.7	2.9	p= <0.001**		
**Myelodysplastic Syndrome (MDS) Patients who have received HSCT**	**All Cause Admissions or Non-COVID-19 Admissions (%)**			**COVID-19 Admissions (%)**		
Mortality rate	5.1	4.0	6.5	17.4	6.7	38.2
**COVID-19 Risk in Myelodysplastic Syndrome (MDS) Patients who have received HSCT**	**Adjusted Odds Ratio**			**p-value**		
Hospitalization risk	0.9	0.6	1.4	p=0.662***		
Mortality risk	4	1.1	14.3	p=0.032****		

MDS stands for Myelodyspastic syndrome patients, COVID-19 admissions represent MDS patients who were admitted for COVID-19 as the primary diagnosis, All cause Admissions or Non-COVID-19 Admissions represent MDS patients who were admitted for diagnosis other than COVID-19, HSCT stands for Hematopoietic stem cell transplantation.

*Matched for age over 65 years, gender, race and hospital characteristics (size, teaching status, and location), comorbidities and complications (stroke, sepsis, ARDS, acute respiratory failure, acute kidney injury, and acute heart failure), and vasopressor usage and based on multivariate regression adjusted for age (≥65 years), gender, race, hospital characteristics (size, teaching status, and location), complications (stroke, ARDS, acute respiratory failure, sepsis, acute kidney injury, and myocardial infarction) and interventions (vasopressor usage and invasive ventilation).

**Matched for age over 65 years, gender, race and hospital characteristics (size, teaching status, and location), comorbidities and complications (stroke, sepsis, ARDS, acute respiratory failure, acute kidney injury, and acute heart failure), and vasopressor usage and based on multivariate regression adjusted for age (≥65 years), gender, race, hospital characteristics (size, teaching status, and location), complications (stroke, ARDS, acute respiratory failure, sepsis, acute kidney injury, and myocardial infarction) and interventions (vasopressor usage and invasive ventilation).

***Matched for age over 65, gender and race based on multivariate regression adjusted with age (≥65 years), gender, race, and hospital characteristics (size, teaching status, and location).

****Matched for age over 65, gender and race based on multivariate regression adjusted for age(≥65 years), gender, race, and hospital characteristics (size, teaching status, and location).

The mortality rate due to COVID-19 (19.6%; 95% CI 17%-22.6%) was higher than non-COVID-19 admissions (6.6%; 95% CI 6.3%-7%). Patients aged ≥65 years had a higher mortality rate (Nonsurvivors 90.1% vs Survivors 88.2%, p=0.5107, non-significant). Male patients also experienced a high mortality rate (Nonsurvivors 64.9% vs Survivors 53.9%, p=0.015). Additionally, among those with a median household income of $65,000 - $85,999, the mortality was high (28.5% vs. 25%, p=0.7966, non-significant). **Table 2** represents Comorbidities that occurred in Acute Myeloid Leukemia and Myelodysplastic Syndrome (MDS) patients with COVID-19.

We observed a significantly higher prevalence of stroke (2% vs 0.3%, p=0.0227), severe sepsis (17.9% vs 0.6%, p<0.0001), acute respiratory distress syndrome (21.8% vs 1.3%, p<0.0001), acute respiratory failure (66.9% vs 49.5%, p=0.0001), and sudden cardiac arrest (10.6% vs 0.2%, p<0.0001) among nonsurvivors. Additionally, the usage of vasopressors (9.3% vs 0.2%, p<0.0001) was markedly higher among nonsurvivors.

After matching the patients based on age, gender, and race and conducting a multivariate regression analysis, which was adjusted for age over 65 years, gender, race, hospital characteristics (size, teaching status, and location), comorbidities and complications (stroke, sepsis, ARDS, acute respiratory failure, acute kidney injury, and acute heart failure), and interventions (vasopressor usage and HSCT), we found an increased risk of mortality due to COVID-19 with an aOR 2.2 (95% CI 1.7-2.9; p<0.001) compared to non-COVID-19 admissions. Following this, we identified several predictors of mortality, such as female gender (aOR=0.6, 95% CI 0.4-1.0, p=0.031), severe sepsis (aOR=9.9, 95% CI 3.3-30, p<0.001), ARDS (aOR=35.1, 95% CI 12.00-102.4, p<0.001), acute respiratory failure (aOR=4.2, 95% CI 2.5-7.00, p<0.001), and vasopressor usage (aOR=7.7, 95% CI 1-60.6, p=0.051). While Age ≥65 years, stroke, Acute Kidney Injury, and Acute Heart Failure did not contribute to mortality. **Table 6** represents the results of the multivariate regression analysis used to study the predictors of mortality in Myelodysplastic syndrome patients with COVID-19.

**Table 6:** Multivariate Logistic Regression to determine predictors of mortality in patients with Myelodysplastic Syndrome and COVID-19.

Predictors of Mortality		95% Confidence Interval		
	Adjusted Odds Ratio	Lower Limit CI	Upper Limit CI	p-value
**Age ≥65 years**	2.1	0.9	4.9	0.080
**Female Gender**	0.6	0.4	1.0	0.031
**Stroke**	3.9	0.9	15.9	0.062
**Severe Sepsis**	9.9	3.3	29.5	**<0.001**
**ARDS**	35.1	12.0	102.4	**<0.001**
**Acute Respiratory Failure**	4.2	2.5	7.0	**<0.001**
**Acute Kidney Injury**	1.4	0.9	2.2	0.166
**Acute Heart Failure and Acute Pulmonary Edema**	1.5	0.9	2.6	0.103
**Vasopressor**	7.7	1.0	60.6	**0.051**
**HSCT status**	1.1	0.3	4.2	0.942

On analyzing the outcomes in MDS patients post-HSCT, we found that 3% were hospitalized for COVID-19, while 3.9% had non-COVID-19 admissions. HSCT did not alter the risk of COVID-19-related hospitalization (aOR 0.9; 95% CI 0.6-1.4; p=0.662, non-significant) when adjusted for age over 65 years, gender, race, and hospital characteristics (size, teaching status, and location). The mortality rate for COVID-19 was 17.4% (95% CI 6.7%-38.2%) while for non-COVID-19 was 5.1% (95% CI 4%-6.5%). After matching the patients based on age over 65, gender, and race and conducting a multivariate regression analysis, adjusted for age over 65 years, gender, race, and hospital characteristics (size and location), we found that COVID-19 was associated with an increased risk of mortality in this population (aOR 4; 95% CI 1.1-14.3; p=0.032) compared to non-COVID-19 admissions.

**Table 4** shows hospital utilization by Acute Myeloid Leukemia (AML) and Myelodysplastic Syndrome (MDS) patients hospitalized for COVID-19. The length of stay and hospital charges were calculated by matching based on age, gender, and race and adjusted for age, gender, race, and hospital characteristics (size, teaching status, and location). COVID-19 patients had a longer stay with a Coefficient of 1.26 days (95% CI 0.67-1.85 days); p<0.001 than non COVID-19 related admissions. The hospital charges did not differ between the two groups (Coefficient $6,230; $-12,405 to $24,865; p=0.512,non-significant).

## Discussion

Our study investigated mortality rates among patients with Acute Myeloid Leukemia (AML) and Myelodysplastic Syndrome (MDS) who were hospitalized for COVID-19. We found that both AML and MDS patients had significantly higher mortality rates compared to the rest of the hospital population. We demonstrated that severe sepsis (aOR of 6.7 in AML; aOR of 9.9 in MDS), acute respiratory distress syndrome (aOR of 17 in AML; aOR of 35.1 in MDS), and acute respiratory failure (aOR of 4.5 in AML; aOR of 4.2 in MDS) had higher mortality with a significant p-value of less than 0.05. Notably, socioeconomic factors such as household income, insurance model, and hospital size did not contribute to mortality. Additionally, patients who underwent hematopoietic stem cell transplantation (HSCT) also had higher mortality rates with COVID-19 in both malignancies.

We found AML patients had higher COVID-19 mortality in those above 65 years compared to survivors (74% vs 48.1%, p=0.0001). This could be attributed to a relatively lower immune status with increasing age and factors like more than three comorbidities (non-survivors 72.6% vs survivors 66.7%). A study conducted by the European Hematology Association Survey (EPICOVIDEHA), also supports our findings in which they found patients with more comorbidities had higher mortality (7). Mortality in male AML patients with COVID-19 was higher than in females but the results did not attain statistical significance (60.3% vs 39.7%, p=0.0989). Jin et al. also reported males in the general population had 2.4 times higher mortality from COVID-19 compared to females of the same age groups (23). The reason for this gender disparity is not fully understood but ACE2 expression is higher in males than in females (23). We found that HSCT had increased COVID-19 hospitalizations (aOR 2.6; 95% CI 1.9-3.4; p<0.001) and increased mortality (aOR 4.1; 95% CI 2-8.7; p<0.001) in AML patients compared to patients admitted for other reasons. Leukemic patients post-HSCT struggle to develop immunity following both natural illness and vaccination (24). We encourage future research in this area, to get a better understanding of the effects of HSCT in COVID-19.

Among patients with Myelodysplastic Syndrome and COVID-19, males had higher mortality than females (64.9% vs 35.1%, p=0.015). This could be due to the same reason as seen in AML patients, i.e. higher protein expression of ACE2 receptors particularly in males, correlating to organ failures (23). We found the in-hospital mortality for MDS was 19.6% (95% CI 17%-22.6%), whereas Fernandez-Cruz et al. reported 38% in those with hematological malignancies (p=0.002), with only 14% of the study population having MDS (25). Complications such as severe sepsis (17.9%), acute kidney injury (45.7%), acute heart failure (25.8%), and ARDS (21.8%) were significantly higher in those with mortality (p<0.0001). COVID-19 can cause endothelial injury, microthrombus formation, and pulmonary cell hyperplasia (26). Complications such as ARDS due to direct viral damage, neutropenia, or immunosuppression increase the risk of sepsis, and a hypercoagulable state due to both viral inflammation and malignancy have led to severe outcomes in MDS patients (25, 27–30). COVID-19 is associated with lower gamma globulin levels and patients with hematological malignancies are prone to have neutropenia and immature granulocytosis. Both these factors lead to increased mortality in this cohort (31, 32). Vasopressors and mechanical ventilation were commonly administered to non-surviving MDSCoV patients, as evidenced by the higher mortality rates among them (nonsurvivors 9.3% vs. survivors 0.2%, p<0.0001). This trend aligns with findings from a study which concluded that critically ill patients with COVID-19 requiring vasopressors were associated with significantly higher mortality (33). Invasive mechanical ventilation was also used in nonsurvivors (3.3%, p<0.0001). However, due to our study design, we couldn’t ascertain the duration of mechanical ventilation or if the patients developed ventilator-associated lung injury (34). We identified that 3% of post-HSCT patients were hospitalized for COVID-19, and 17.4% of those did not survive. This mortality rate is higher compared to post-HSCT patients hospitalized for other reasons (5.1%). HSCT has been shown to retain anti-SARS-CoV-2 IgG antibodies in patients with hematological malignancies (35). However, the higher mortality rate in this subgroup warrants investigation.

Our research faced several limitations, primarily due to its design. The values we reported pertain only to the hospitalized population and the exact prevalence of COVID-19 from the general population could not be determined. We were unable to follow up with survivors or compare results across different waves of COVID-19. Additionally, laboratory results of patients at admission were unavailable, preventing us from determining or monitoring trends in various complications. Furthermore, we could not account for the newer management guidelines that have evolved since the start of the pandemic, nor did we account for coding errors and duplication of data.

## Conclusion

In conclusion, our study indicates that AML and MDS patients with COVID-19, have higher inpatient mortality with sepsis and respiratory complications emerging as significant contributors to this outcome. Additionally, patients post-HSCT had higher mortality in COVID-19 within both AML and MDS. These findings underscore the need for close monitoring and implementation of preventive strategies. Future research is warranted to identify optimal treatment approaches and risk mitigation strategies specifically for AMLand MDS patients.

## Data Availability

The datasets presented in this study can be found in online repositories. The names of the repository/repositories and accession number(s) can be found in the article/**Supplementary Material**.

## Supplementary material

Supplementary Table 1 ICD 10 Codes used in the making of the study -This table includes all the ICD codes that were used in our study.
