# Supplementary material for: COVID-19 in acute myeloid leukemia (AML) and myelodysplastic syndrome (MDS): a propensity matched analysis (2020-2021)
Source: Front Oncol. 2024 Oct 17;14:1446482. doi: 10.3389/fonc.2024.1446482 (PMC11524996; doi:10.3389/fonc.2024.1446482)
Supplement: Supplementary file 2 [file Table2.docx]

**Supplementary file table 2: Sociodemographic Profile of Acute Myeloid Leukemia Patients**

| **Acute Myeloid Leukemia (n=28,028)** | | | | **Acute Myeloid Leukemia with COVID-19 (n=336)** | | |
| --- | --- | --- | --- | --- | --- | --- |
|  | **Without COVID-19 Percentage (%)** | **With COVID-19 Percentage (%)** | **p-value** | **Survival**  **Percentage (%)** | **Mortality Percentage (%)** | **p-value** |
| **Age ≥65 years** | 51.1 | 53.7 | 0.333 | 48.1 | 74 | **0.0001** |
| **Gender** |  |  | 0.3676 |  |  | 0.1028 |
| Male | 54.3 | 51.8 |  | 49.4 | 60.3 |  |
| Female | 45.7 | 48.2 |  | 50.6 | 39.7 |  |
| **Race** |  |  | **0.0186** |  |  | 0.4637 |
| White | 72.1 | 65.7 |  | 63.6 | 73.2 |  |
| Black | 10.2 | 13.3 |  | 13 | 14.1 |  |
| Hispanic | 9.8 | 13.6 |  | 14.6 | 9.9 |  |
| Asian or Pacific Islander | 4.3 | 2.7 |  | 3.1 | 1.4 |  |
| Native American | 0.4 | 0.9 |  | 1.1 | 0 |  |
| Other | 3.3 | 3.9 |  | 4.6 | 1.4 |  |
| **Median Household Income** |  |  | **0.0011** |  |  | 0.5907 |
| $1 - $49,999 | 23.0 | 28.6 |  | 27.3 | 33.3 |  |
| $50,000 - $64,999 | 24.8 | 30.4 |  | 31.9 | 25 |  |
| $65,000 - $85,999 | 25.4 | 21.1 |  | 21.5 | 19.4 |  |
| $86,000 or more | 26.7 | 19.9 |  | 19.2 | 22.2 |  |
| **Primary Payer** |  |  | 0.377 |  |  | - |
| Medicare | 49.6 | 54.3 |  | 50.4 | 68.5 |  |
| Medicaid | 12.1 | 13.4 |  | 16.3 | 2.7 |  |
| Private including HMO | 32.9 | 29.4 |  | 30.7 | 24.7 |  |
| Self-pay | 1.8 | 0.9 |  | 1.1 | 0 |  |
| No charge | 0.3 | 0.0 |  | 0 | 0 |  |
| Other | 3.2 | 2.1 |  | 1.5 | 4.1 |  |
| **Census Division of hospital** |  |  | 0.1941 |  |  | **0.0138** |
| New England | 4.9 | 3.9 |  | 2.6 | 8.2 |  |
| Mid-Atlantic | 14.1 | 16.0 |  | 14.8 | 20.5 |  |
| East North Central | 14.9 | 17.5 |  | 17.8 | 16.4 |  |
| West North Central | 7.6 | 7.1 |  | 8.7 | 1.4 |  |
| South Atlantic | 20.0 | 22.0 |  | 23.9 | 15.1 |  |
| East South Central | 6.4 | 5.9 |  | 4.2 | 12.3 |  |
| West South Central | 11.8 | 9.5 |  | 9.1 | 11 |  |
| Mountain | 6.0 | 8.0 |  | 9.1 | 4.1 |  |
| Pacific | 14.2 | 10.1 |  | 9.9 | 11 |  |
| **Hospital size according to the number of beds** |  |  | 0.1689 |  |  | 0.5683 |
| Small | 13.2 | 16.3 |  | 17.4 | 12.3 |  |
| Medium | 18.5 | 19.9 |  | 19.3 | 21.9 |  |
| Large | 68.3 | 63.8 |  | 63.3 | 65.7 |  |
| **Location/ teaching status of hospital** |  |  | **<0.0001** |  |  | 0.2692 |
| Rural | 3.0 | 6.8 |  | 6.4 | 8.2 |  |
| Urban non-teaching | 7.9 | 15.4 |  | 17.1 | 9.6 |  |
| Urban teaching | 89.1 | 77.7 |  | 76.5 | 82.2 |  |
| **Hospital Region** |  |  | 0.6805 |  |  | **0.1352** |
| Northeast | 19.1 | 19.9 |  | 17.4 | 28.8 |  |
| Midwest | 22.5 | 24.6 |  | 26.5 | 17.8 |  |
| South | 38.2 | 37.4 |  | 37.1 | 38.4 |  |
| West | 20.2 | 18.1 |  | 18.9 | 15.1 |  |
| **Charlson Comorbidity Index** |  |  | **-** |  |  | - |
| 2 comorbidities | 40.9 | 32.1 |  | 33.3 | 27.4 |  |
| ≥3 comorbidities | 59.1 | 67.9 |  | 66.7 | 72.6 |  |
| **Disposition of patients** |  |  | **<0.0001** | - | - | **-** |
| Routine | 54.4 | 47.5 |  |  |  |  |
| Transfer to a short-term hospital | 4.3 | 3.0 |  |  |  |  |
| Transfer, including skilled nursing facility, intermediate care, and other types of facility | 8.1 | 9.8 |  |  |  |  |
| Home health care | 23.9 | 17.2 |  |  |  |  |
| Against medical advice | 0.7 | 0.6 |  |  |  |  |
| Discharged alive, destination unknown | 0.0 | 0.3 |  |  |  |  |
| **Died in hospital** | 8.5 | 21.7 | **<0.0001** | - | - | **-** |

**Supplementary file table 3: Sociodemographic Profile of Patients with Myelodysplastic Syndrome**

| **Myelodysplastic Syndrome (n=28,256)** | | | | **Myelodysplastic Syndrome with COVID-19 (n=769)** | | |
| --- | --- | --- | --- | --- | --- | --- |
|  | **Without COVID-19 Percentage (%)** | **With COVID-19**  **Percentage (%)** | **p-value** | **Survival Percentage (%)** | **Mortality Percentage (%)** | **p-value** |
| **Age ≥65 years** | 84.5 | 88.6 | **0.0026** | 88.2 | 90.1 | 0.5107 |
| **Gender** |  |  | 0.855 |  |  | **0.015** |
| Male | 56.4 | 56 |  | 53.9 | 64.9 |  |
| Female | 43.6 | 44 |  | 46.1 | 35.1 |  |
| **Race** |  |  | 0.1079 |  |  | 0.5267 |
| White | 79.1 | 76.4 |  | 77.3 | 72.8 |  |
| Black | 8.8 | 10.9 |  | 10.1 | 14.3 |  |
| Hispanic | 6.7 | 7.9 |  | 7.5 | 9.5 |  |
| Asian or Pacific Islander | 3.1 | 2 |  | 2 | 2 |  |
| Native American | 0.3 | 0.5 |  | 0.7 | 0 |  |
| Other | 2 | 2.3 |  | 2.5 | 1.4 |  |
| **Median Household Income** |  |  | 0.174 |  |  | 0.7966 |
| $1 - $49,999 | 22.7 | 25.1 |  | 25.5 | 23.8 |  |
| $50,000 - $64,999 | 25.3 | 25.5 |  | 25.5 | 25.8 |  |
| $65,000 - $85,999 | 24.9 | 25.7 |  | 25 | 28.5 |  |
| $86,000 or more | 27 | 23.7 |  | 24.1 | 21.8 |  |
| **Primary Payer** |  |  | **0.0151** |  |  | 0.4117 |
| Medicare | 80.7 | 85.3 |  | 85.3 | 85.4 |  |
| Medicaid | 3.9 | 2.6 |  | 2.8 | 2 |  |
| Private including HMO | 12.3 | 9 |  | 8.9 | 9.3 |  |
| Self-pay | 0.8 | 0.4 |  | 0.2 | 1.3 |  |
| No charge | 0.1 | 0.1 |  | 0.2 | 0 |  |
| Other | 2.2 | 2.6 |  | 2.8 | 2 |  |
| **Census Division of hospital** |  |  | **0.0123** |  |  | 0.4401 |
| New England | 6 | 4.9 |  | 4.9 | 5.3 |  |
| Mid-Atlantic | 16.2 | 18.3 |  | 17.5 | 21.8 |  |
| East North Central | 15.9 | 17.8 |  | 18.6 | 14.6 |  |
| West North Central | 7.3 | 8.8 |  | 8.6 | 9.9 |  |
| South Atlantic | 21.7 | 21.5 |  | 21.8 | 19.9 |  |
| East South Central | 5 | 4.5 |  | 5 | 2.6 |  |
| West South Central | 9.1 | 10.3 |  | 10.4 | 9.9 |  |
| Mountain | 5 | 4.7 |  | 5 | 3.3 |  |
| Pacific | 13.7 | 9.1 |  | 8.3 | 12.6 |  |
| **Hospital size according to the number of beds** |  |  | **0.0014** |  |  | 0.6615 |
| Small | 21.1 | 26 |  | 26.5 | 23.8 |  |
| Medium | 27.3 | 28.5 |  | 27.8 | 31.1 |  |
| Large | 51.6 | 45.5 |  | 45.6 | 45 |  |
| **Location/ teaching status of hospital** |  |  | **<0.0001** |  |  | 0.4237 |
| Rural | 7.2 | 12.7 |  | 12.5 | 13.9 |  |
| Urban non-teaching | 17.7 | 19 |  | 19.9 | 15.2 |  |
| Urban teaching | 75.1 | 68.3 |  | 67.6 | 70.9 |  |
| **Hospital Region** |  |  | **0.0044** |  |  | 0.3941 |
| Northeast | 22.2 | 23.3 |  | 22.3 | 27.2 |  |
| Midwest | 23.2 | 26.7 |  | 27.2 | 24.5 |  |
| South | 35.9 | 36.3 |  | 37.2 | 32.5 |  |
| West | 18.7 | 13.8 |  | 13.3 | 15.9 |  |
| **Charlson Comorbidity Index** |  |  | **0.0071** |  |  | 0.6539 |
| 0 comorbidities | 12.7 | 12.6 |  | 13.1 | 10.6 |  |
| 1 comorbidity | 14.8 | 17.7 |  | 18.1 | 15.9 |  |
| 2 comorbidities | 17.8 | 20.8 |  | 20.9 | 20.5 |  |
| ≥3 comorbidities | 54.7 | 48.9 |  | 47.9 | 53 |  |
| **Disposition of patients** |  |  | **<0.0001** | - | - | **-** |
| Routine | 40.9 | 32 |  |  |  |  |
| Transfer to a short-term hospital | 2.4 | 1.6 |  |  |  |  |
| Transfer, including skilled nursing facility, intermediate care, and other types of facility | 21.1 | 21.6 |  |  |  |  |
| Home health care | 28.2 | 24.7 |  |  |  |  |
| Against medical advice | 0.7 | 0.4 |  |  |  |  |
| Discharged alive, destination unknown | 0 | 0.1 |  |  |  |  |
| **Died in hospital** | 6.6 | 19.6 | **<0.0001** | - | - | **-** |

**Supplementary file Table 4: Univariate Logistic Regression of AML patients and COVID-19 who did not survive**

| **AML patients with COVID who expired (n=73)** | | | | |
| --- | --- | --- | --- | --- |
| **Mortality Predictors** | | **95% CI** | |  |
|  | **Odds ratio** | **Lower limit CI** | **Upper limit CI** | **p-value** |
| **Age ≥65 years** | 3.07 | 1.75 | 5.38 | **<0.001** |
| **Male Gender** | 1.56 | 0.92 | 2.65 | 0.098 |
| **Location/ teaching status of hospital (Ref=Rural)** |  |  |  |  |
| Urban non-teaching | 0.44 | 0.13 | 1.48 | 0.184 |
| Urban teaching | 0.84 | 0.31 | 2.27 | 0.733 |
| **Hospital Region (Ref=Northeast)** |  |  |  |  |
| Midwest | 0.41 | 0.18 | 0.94 | **0.036** |
| South | 0.63 | 0.31 | 1.28 | 0.199 |
| West | 0.48 | 0.20 | 1.18 | 0.109 |
| **Stroke** | 19.34 | 2.23 | 168.02 | 0.007 |
| **Severe sepsis** | 18.23 | 6.49 | 51.21 | <0.001 |
| **ARDS** | 12.08 | 5.02 | 29.08 | <0.001 |
| **Acute Respiratory Failure** | 1.75 | 1.01 | 3.05 | 0.047 |
| **Acute Kidney Injury** | 2.81 | 1.60 | 4.93 | <0.001 |
| **Arrhythmia (Ventricular fib, Ventricular tachycardia, Atrial fib, Atrial flutter)** | 1.64 | 0.65 | 4.18 | 0.296 |
| **Acute Heart Failure and Acute Pulmonary Edema** | 2.06 | 0.74 | 5.76 | 0.168 |
| **Myocardial Infarction** | 9.14 | 2.71 | 30.79 | <0.001 |
| **Blood Transfusion** | 2.21 | 1.16 | 4.21 | 0.015 |
| **Vasopressor** | 18.85 | 5.24 | 67.81 | <0.001 |
| **Invasive Ventilation** | 7.41 | 0.67 | 81.78 | 0.102 |

**Supplementary file Table 5: Univariate Logistic Regression of MDS patients and COVID-19 who did not survive**

| **MDS patients with COVID who expired (n=151)** | | | | |
| --- | --- | --- | --- | --- |
| **Mortality Predictors** | | **95% CI** | |  |
|  | **Odds ratio** | **Lower limit CI** | **Upper limit CI** | **p-value** |
| **Male Gender** | 1.58 | 1.09 | 2.3 | **0.016** |
| **Stroke** | 6.24 | 1.03 | 37.75 | **0.046** |
| **Severe sepsis** | 33.42 | 11.49 | 97.26 | **<0.001** |
| **ARDS** | 21.32 | 9.56 | 47.58 | **<0.001** |
| **Acute Respiratory Failure** | 2.06 | 1.41 | 3 | **<0.001** |
| **Acute Kidney Injury** | 2.05 | 1.42 | 2.96 | **<0.001** |
| **Acute Heart Failure and Acute Pulmonary Edema** | 1.8 | 1.18 | 2.76 | **0.007** |
| **Myocardial Infarction** | 2.67 | 1.44 | 4.96 | **0.002** |
| **Sudden Cardiac Arrest** | 73.12 | 9.62 | 556.09 | **<0.001** |
| **Diabetes** | 0.7 | 0.47 | 1.03 | 0.072 |
| **Vasopressor** | 63.05 | 8.22 | 483.8 | **<0.001** |
